# Stem cell-derived exosomal transcriptomes for wound healing

**DOI:** 10.3389/fsurg.2022.933781

**Published:** 2022-08-12

**Authors:** Guiling Chen, Hankun Chen, Xiang Zeng, Wei Zhu

**Affiliations:** ^1^ The Second Clinical College of Guangzhou University of Chinese Medicine, Guangzhou, China; ^2^ The Second Affiliated Hospital of Guangzhou University of Chinese Medicine, Guangzhou, China; ^3^ National Institute of Stem Cell Clinical Research, Guangdong Provincial Hospital of Chinese Medicine, Guangzhou, China; ^4^ Guangdong Provincial Academy of Chinese Medical Sciences, Guangzhou, China; ^5^ Research and Development Department, Guangzhou Qinglan Biotechnology Company Limited, Guangzhou, China

**Keywords:** wound healing, stem cell, exosome, microRNA, long noncoding RNA, circular RNA, messenger RNA

## Abstract

Wound healing is a complex and integrated process of the interaction of various components within the injured tissue. Accumulating evidence suggested that stem cell-derived exosomal transcriptomes could serve as key regulatory molecules in wound healing in stem cell therapy. Stem cell-derived exosomal transcriptomes mainly consist of long noncoding RNAs (lncRNAs), microRNAs (miRNAs), circular RNAs (circRNAs) and messenger RNAs (mRNAs). In this article we presented a brief introduction on the wound repair process and exosomal transcriptomes. Meanwhile, we summarized our current knowledge of the involvement of exosomal transcriptomes in physiological and pathological wound repair process including inflammation, angiogenesis, and scar formation.

## Introduction

Skin wounds are classified primarily into acute and chronic wounds. Acute wounds included cuts, scrapes, burns, trauma, needle punctures, and surgical incisions. While chronic wounds refer to wounds like diabetic foot ulcers, pressure ulcers and infected wounds (1). The healing of wound is the process of tissue regeneration involving the synergistic and integrated actions of cells and intercellular factors, cells and cells, cells and the microenvironment. The normal wound healing involves four successive but overlapping phases, including the hemostasis phase, the inflammatory phase, the proliferative phase, and the remodeling phase (2). Many factors affect and interfere with normal wound healing, such as infection, medication, complex comorbidities, age, obesity, smoking and nutrition (3, 4). Any disturbance in these four phases affects wound healing or even proper functioning of the skin, results in the formation of chronic non-healing wounds or repaired with hypertrophic scar tissue or keloids (5).

Currently, therapeutic research on wound healing is focused on the use of small molecules, including growth factors, insulin, stromal cell-derived factor-1, antimicrobial peptides, platelet rich plasma, and so on (6). In addition, silk biomaterials, artificial skin grafts, honey are also potential therapeutic means for wound healing (7–9). In recent decades, stem cell therapy has become a promising approach in wound healing due to its distinguished functions on tissue regeneration, immunoregulation and angiogenesis (10). Stem cells participate in the regulation of inflammatory, proliferative and remodeling phases *via* paracrine of cytokines, chemokines and growth factors. Stem cells ranging from immature pluripotent stem cells to more restricted multipotent progenitor cells have been investigated for their abilities in facilitating wound healing in several animal models and clinical trials (11). However, because stem cells do not readily survive in the wound microenvironment in a large portion of cases, their effects in wound healing may be difficult to be accomplished (12). One fundamental hurdle is that reactive oxygen species can weaken stem cell function and therefore impair wound healing (13). In addition, small molecules are easily degraded in wound fluids and carry risks of drug resistance, new therapies are still being explored.

Stem cell-derived exosomes have demonstrated great potentials to provide therapeutical benefits in nearly all stages of wound healing process, including alleviating inflammation (14), promoting vascularization (15), promoting proliferation and migration of epithelial cells and fibroblasts (16), while reducing scar formation (17). However, the exact mechanism of action remains to be explored. Growing evidences have shown that exosome-derived transcriptomes (RNAs) participate in comprehensive biological and functional aspects of physiological conditions (18–20). Therefore, stem cell derived exosomal transcriptomes attracted great research interests (21–24). In this review, we summarized the process of wound healing and several key RNAs of exosome transcriptomes involved, such as messenger RNAs (mRNAs), microRNAs (miRNAs), long non-coding RNAs (lncRNAs) and circular RNAs (circRNAs), with the focus on the effects of several transcriptional molecules of stem cell derived exosomes on wound healing.

## Wound healing process

Wound healing is a complex and dynamic process to skin injury. Wound healing is classically divided into four overlapping phases: hemostasis, inflammation, proliferation and remodeling. During the hemostatic phase, platelets and circulating coagulant factors accumulate and interact at the site of tissue injury, followed by activation of endothelial cells. Inflammation state involves complex mechanisms among resident immune cells, circulating leucocytes and their pro-inflammatory cytokines and chemokines. Uncontrolled and excessive inflammation promotes tissue injury and delays healing, thereby causing chronic wounds or scars. The proliferative phase of healing is characterized by extensive activation of keratinocytes, epidermal stem cells, fibroblasts, macrophages and endothelial cells to orchestrate wound closure and angiogenesis. Extracellular matrix (ECM) remodeling spans the entire wound healing process. Fibroblasts, myofibroblasts, collagen, transforming growth factor (TGF)-β, proteoglycans and matrix metalloproteinases are major players involved in ECM remodeling, balancing collagen synthesis and degradation (25, 26).

Disturbance of any of the above stages of the normal wound healing process can lead to pathological wound healing, which mainly includes two types: excessive scarring and chronic wound healing. Persistent activation of TGF-β pathway provided a deviant signal to myofibroblasts, leading to continuous production of ECM triggering pathological scarring (27). Hyper-proliferation of fibroblasts and excessive collagen deposition may lead to hypertrophic scar formation as well (28). Chronic wounds are typically caused by a temporal arrest in the inflammation phase with no further progress to the stages of healing. Chronic wounds are characterized by massive immune cell infiltration, excessive levels of proinflammatory cytokines, persistent infection, the formation of biofilms of drug-resistant microorganisms, and appearance of senescent cells which are unresponsive to restorative stimuli (29, 30).

## Exosomes and exosomal transcriptomes

Exosomes are bioactive membranous vesicles with a diameter of 40–100 nm secreted by many type of cells. They were discovered for the first time in sheep reticulocytes in 1983 (31). Exosome formation begins with endocytosis on the surface of the cell membrane with the formation of early endosomes *via* inward budding (32). Recently, various nucleic acids have been identified within exosomes, including mRNAs and non-coding RNAs such as miRNAs, lncRNAs, circRNAs, ribosomal RNAs (rRNAs), transfer RNAs (tRNAs), small nucleolar RNAs (snoRNAs), small nuclear RNAs (snRNAs) and piwi-interacting RNAs (piRNAs). These RNAs are transferred from parent cells to recipient cells through exosomes to exert specific functions (33, 34). Secreted exosomes can be transferred to recipient cells *via* endocytosis, direct membrane fusion, and receptor ligand interactions. Exosomes are associated with a wide spectrum of physiological and pathological processes including but not limited to immune responses, viral pathogenicity, pregnancy, cardiovascular diseases, central nervous system-related diseases, cancer progression and so on. In addition, exosomes have potential as diagnostic and therapeutic tools for a variety of diseases, including neurodegenerative diseases, cardiovascular dysfunction, and cancer (35).

Exosomal transcriptomes mainly contain mRNAs, lncRNAs, miRNAs and circRNAs, which have been intensively studied in a number of studies. mRNAs, macromolecules carry codes from the DNA in the nucleus to the cytoplasm (the ribosomes), where protein synthesis occurs, were first found highly enriched in exosomes released by mast cells (36, 37). mRNAs in exosomes differ significantly from those in the cytoplasm of the donor cells (37). Exosomal mRNAs could be transferred to other cells and be translated into proteins in the target cells (37, 38). Exosomes derived from cells growing under oxidative stress may induce tolerance of the recipient cells to external stress by mRNAs transported by exosomes (39). In addition, exosomal mRNAs may potentially play a role in epigenetic inheritance in mammals (40).

**Figure 1:**
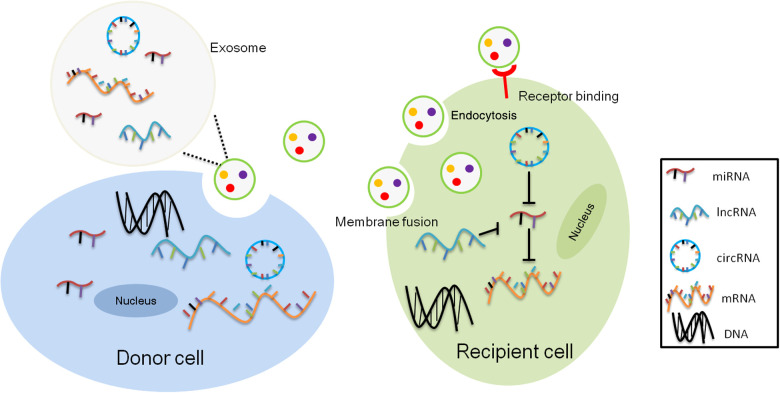
Intercellular transcriptomes delivery by exosomes. Exosomal noncoding RNAs, mainly microRNAs (miRNAs), long noncoding RNAs (lncRNAs), and circular RNAs (circRNAs) from donor cells act as competitive endogenous RNAs (ceRNAs) for binding to common miRNA response elements (MREs) from recipient cells altering their activity.

Although 70% of the human genome is transcribed into RNAs, only 2% of these transcripts are translated into proteins. The remaining transcripts are defined as noncoding RNAs, including lncRNAs, miRNAs and circRNAs, which participate in the regulation of mRNA and protein function, in the binding with DNA to modulate gene transcription, or in acting as the competing endogenous RNA (ceRNA) or miRNA sponges to regulate gene expression (Figure 1). LncRNAs are a category of cellular RNAs which are longer than 200 nucleotides in length but in lack of the open reading frames. This class comprises, among others, long intergenic noncoding RNAs (lincRNAs), antisense RNAs, and sense overlapping lncRNAs. LncRNAs may serve as biomarkers of cancer, such as nuclear enriched abundant transcript 1 (NEAT1), HOX Transcript Antisense RNA (HOTAIR), metastasis-associated lung adenocarcinoma transcript 1 (MALAT-1), H19. Moreover, lncRNAs participated in cancer development, progression, metastasis and prognosis (41), and other diseases like cerebrovascular diseases, cardiac diseases, inflammatory bowel diseases, metabolic syndrome related disorders as well (42–45).The abundance of exosomal lncRNAs correlates with their expression levels in the cell of origin (46).

Among the RNAs in exosomes, miRNAs have attracted the most attention due to their roles in gene expression regulation. MiRNAs are a class of 17–24 nt, small, noncoding and evolutionarily conserved RNAs, They can mediate post-transcriptional gene silencing by targeting the 3′-UTR of target mRNAs under the control of the RNA-induced silencing complex (47–49). MiRNAs can regulate cell proliferation and differentiation (50), and thus participate in development (51, 52). They can also serve as therapeutic targets and prognostic markers for diseases and cancers (53, 54).

CircRNAs are a new category of single-stranded RNAs characterized by their covalently closed loop structures lacking of 5′ caps or a 3′ poly(A) tails in all eukaryotic cells. They are generated through a particular mechanism of alternative splicing called “back-splicing”. In 2015, Li et al. reported for the first time that exosomes contained abundant circRNAs. Genome-wide RNA-seq analysis found that circRNAs were enriched in the exosomes relative to the parental cells. It has been reported that the sorting of circRNAs to exosomes can be controlled by modulating the levels of relevant miRNAs in parental cells and can transfer bioactive substance to target cells (55). It has been confirmed that circRNAs could exert biological functions in multiple aspects, including participating in neuronal development (56) and the pathogenesis of cancer, cardiovascular, neurological, and autoimmune diseases (57–60). In addition, circRNAs serve as potential biomarkers for many diseases (61), especially cancer (62).

## Advantages of exosomes and exosomal transcriptomes in wound healing

Harsh wound microenvironments, such as deficiency of nutritional factors, enhanced inflammatory response, increased reactive oxygen species, and impaired vasculature, all resulted in poor survival of the transplanted stem cells (63).

Stem cell derived exosomes carrying bioactive contents of parental stem cells, transmit signals and participate in the remodeling of extracellular matrix through paracrine mechanism. Exosomes are less immunogenic, more stable and easier to store relative to their donor stem cells, and therefore can be used as an alternative therapy for stem cells in wound healing regulation. There have been clinical trials on stem cell derived exosomes for the treatment of diabetic wounds registered on the Clinicaltrial.gov website [NCT05243368]. Besides, patents on stem cell derived exosomes as wound dressings have been published [CN114376989A&CN114377194A]. However, the low yields of stem cell derived exosomes require a large number of stem cells to be consumed. Exosome components are complex and can hardly be purified due to the limitation of extraction techniques. The transcriptomes of exsomes, however, as intercellular transport cargos of exosomes, is relatively well-defined in composition and more suitable for research. Moreover, their functions in wound healing can be defined by loss- or gain-of-function experiments after overexpression or knockdown their expressions in the parent cells. Unlike the great risks such as mutagenesis, toxicity, and limited capacity for genetic cargo posed by viral vectors, exosomal RNAs transferring presented good safety and biocompatibility (64).

At present, research on stem cell derived exosomes mainly focuses on those derived from mesenchymal stem cells (65–67). However, there are still some studies on exosomes from other sources of stem cells, such as pluripotent stem cells (68, 69) or embryonic stem cells (70). Consistent with the major functions of the parental cells, exosomes derived from MSCs embrace immunomodulatory as well as regenerative effects on the recipient cells (21, 71).

## Transcriptomes of stem cell exosomes and wound healing

**Figure 2:**
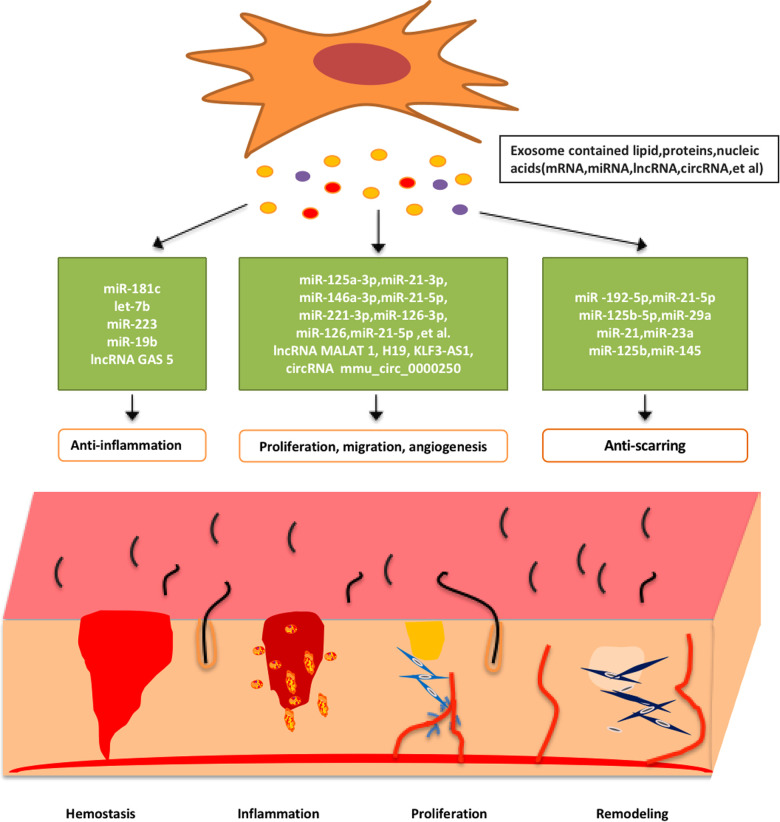
Schematic demonstration of the exosomal RNAs derived from stem cells promote wound healing by enhancing angiogenesis, cell proliferation and migration, reducing inflammation, and anti-scarring.

The stem cell derived exosomal transcriptomes have been shown to promote wound healing in terms of anti-inflammation, cell proliferation and migration, blood vessel formation, and inhibition of scar formation, and the effects on wound healing of four transcriptomes, including miRNAs, lncRNAs, circRNAs, as well as mRNAs, will be illustrated below (see Figure 2).

### Stem cell derived exosomal miRNAs and wound healing

A number of studies have demonstrated that miRNAs were the active molecules of stem cell derived exosomes to regulate the functions of the recipient cells (72, 73). In wound healing for examples, miRNAs in the stem cell derived-exosomes have shown potent effects on regeneration, anti-inflammatory and anti-scarring to the wounds (see Table 1).

**Table 1:** Stem cell-exosome derived miRNA-mediated regulation of wound healing.

Exosome derived miRNAs	Donor cells	Recipient cells	Potential target factor	Functions	References
miR-125a-3p	ADSCs	HUVECs	PTEN↓	Promote cell viability, migration and angiogenesis	(106)
miR-21-3p	Umbilical cord blood	Fibroblasts and endothelial cells	PTEN↓ SPRY1↓	Promote fibroblast proliferation and migration, enhance endothelial cell angiogenic activity	(75)
miR -146a	ADSCs	Fibroblasts	Serpin family H member 1 and p-ERK2↑	Promote the migration and proliferation of fibroblasts, and neovascularization	(74)
miR-21-5p	BMSCs	Fibroblasts and HUVECs	SPRY2↓; PI3K/AKT and ERK1/2↑	Promote the proliferation, migration and angiogenesis of fibroblasts and HUVECs	(77)
miR-221-3p	BMSCs	Endothelial cells	AKT/eNOS↑	Promote endothelial cells proliferation, migration, tube formation and VEGF levels	(107)
miR-126-3p	Synovium mesenchymal stem cells	Fibroblasts and HMEC-1	—	Promote proliferation of human dermal fibroblasts and human dermal microvascular endothelial cells (HMEC-1); Promote g HMEC-1 migration and tube formation Promote collagen maturation	(76)
miR-126	BMSCs	HUVECs	PI3K/AKT ↑	Promote the HUVECs proliferation, migration and angiogenesis	(108)
miR-21-5p and let-7c-5p	BMSCs	HUVECs	–	Enhance cell proliferation rate, migration and tube formation	(109)
miR-200a	Embryonic stem cells	Endothelial cells	Kelch-like ECH- associated protein 1↓ nuclear factor erythroid2-related factor 2↑	Rejuvenate the senescence of endothelial cells and recover compromised proliferation, migratory capacity, and tube formation	(79)
miR-125a	ADSCs	Endothelial cells	DLL4↓	Promote endothelial cell angiogenesis	(110)
miR-21	ADSCs	HaCaT cells	PI3K/AKT ↑	Enhance the migration and proliferation	(111)
miR-135a	Human amnion mesenchymal stem cells	BJ cells	Large tumor suppressor kinase 2↓	Promote cell migration and transformation	(78)
miR-378	ADSCs	HaCaT cells	Caspase-3↑	Promote proliferation and migration and reducing apoptosis	(112)
miR -93-3p	BMSCs	HaCaT cells	Apoptotic peptidase activating factor 1 ↓	Promote proliferation and migration and reducing apoptosis	(113)
miR-100-5p and miR-1246	Stem cells of human deciduous exfoliated teeth	HUVECs	VEGFA↓	Inhibite cell proliferation and migration and inducing apoptosis	(80)
miR-181c	UCMSCs	Macrophages	TLR4↓	Alleviate inflammation	(81)
let-7b	UCMSCs	Macrophages	TLR4/NF-κB/STAT3/AKT↓	Regulate macrophage plasticity and alleviating inflammation	(82)
miR-223	BMSCs	Macrophages	Pknox1↓	Promote macrophage toward M2 polarization	(83)
miR-19b	ADSCs	Fibroblasts	CCL1↓TGF-β↑	Regulate inflammation	(84)
miR -192-5p	ADSCs	Fibroblasts	IL-17RA↓Smad↓	Anti- fibrosis	(87)
miR-21-5p and miR-125b-5p	UCMSCs	Fibroblasts	TGF-β receptor type II and TGF-β receptor type I ↓	Anti-myofibroblast differentiation	(17)
miR-29a	ADSCs	Fibroblasts	TGF-β2/Smad3↓	Inhibit fibrosis and scar hyperplasia of fibroblasts	(86)
miR-21, mir-23a, miR-125b, and miR-145	UCMSCs	Myofibroblasts	TGF-β/SMAD2↓	Suppress α-smooth muscle actin formation and collagen deposition	(88)

ADSC, adipose-derived mesenchymal stem cell; BMSC, bone marrow-derived mesenchymal stem cell; UCMSC, human umbilical cord-derived mesenchymal stem cells. HUVECs, human umbilical vein endothelial cells; HMEC-1, human dermal microvascular endothelial cells; PTEN, phosphatase and tensin homolog; SPRY, sprouty RTK signaling antagonist; TGF-β, transforming growth factor-β; ↑, upregulated; ↓, downregulated.

#### Exosomal miRNAs on skin regeneration

During wound healing process, regeneration is mainly manifested by promoting fibroblasts proliferation, boosting vascularization, formation of granulation tissue, and enhancing re-epithelialization, among other phathophyioloigcal events. Four miRNAs (miR-126-3p, miR-21-3p, miR-146a-3p and miR-21-5p) were reported to have the potentials to enhance the proliferation and migration of the fibroblasts and endothelial cells, and to promote angiogenesis of human endothelial cells through phosphatase and tension homologs (PTEN), PI3K/AKT and ERK signaling pathways, respectively (74–77). Local application of miRNA-21-3p-enriched umbilical cord blood-exosomes into mouse skin wounds accelerated re-epithelialization, reduced scar width, and enhanced angiogenesis. Inhibition of PTEN and sprouty RTK signaling antagonist 1 may be the key function of miR-21-3p (75). MiR-146a-3p, derived from ADSC-exosomes could promote the proliferation and migration of fibroblasts by inducing the expressions of serpin family H member 1 and p-ERK2 to rapidly reduce the wound area and foster the formation of new blood vessels in rats with wounds (74). An *in vitro* study by Tao et al. found that exosomes over-expressing miR-126-3p derived from synovium mesenchymal stem cells stimulated the proliferation of human dermal fibroblasts and human dermal microvascular endothelial cells (HMEC-1) in a dose-dependent manner, and enhanced HMEC-1 migration and tube formation as well. In addition, exosomes overloaded with miR-126-3p accelerated re-epithelialization, activated angiogenesis, and promoted collagen maturation in diabetic wound healing (76). Gao et al. suggested that exosomes enriced in miR-135a from human amniotic mesenchymal stem cells have shown efficacy in enhacing epidermal cell migration to promote wound healing in SD rats. Furthermore, knockdown of miR-135a in ADSC-exosomes validly attenuated the effect of exosomes on BJ fibroblasts migration. The study suggested that the abovementioned function of miR-135a may be realized through inhibiting the expression of large tumor suppressor kinase 2 (78).

MiRNAs from other stem cell derived exosomes have been reported to enhance cell proliferation and migration to improve wound healing. MiR-200a-enriched embryonic stem cell derived exosomes were capble of rejuvenating vascular endothelial cell senescence and restore impaired proliferation, migration, and tube formation by downregulating Keap1 to activated nuclear factor erythroid2-related factor 2 (79).

Although most stem cell-exosomes derived miRNAs can promote cell proliferation, migration, and angiogenesis, a small proportion of miRNAs play a part in inhibiting wound healing. Exosomes enriched with miR-100-5p and miR-1246 from human deciduous tooth exfoliated dental stem cells were found to decrease cell proliferation and migration of HUVECs, induce cell apoptosis, and suppress tube like structure formation of HUVECs by downregulating several angiogenesis related factors, including VEGFA, MMP-9 and angiopoietin 1 (80), suggesting that miR-100-5p and miR-1246 play an anti-angiogenic role in wound healing.

#### Exosomal miRNAs on anti-inflammation

The inflammatory response performs multiple tasks at the wound site by improving wound debridement and producing chemokines, metabolites, and growth factors. If this orchestrated response becomes dysregulated, wounds can become chronic or progressive fibrosis, both of which can impair tissue function and ultimately lead to organ failure and even death. Investigations have shown that miR-181c in UCMSC exosomes played a critical role in regulating burn induced inflammation. Exosomes overexpressing miR-181c potently inhibited toll-like receptor 4 (TLR4) signaling and attenuated inflammation in skin-burned rats, and significantly reduced lipopolysaccharide (LPS) induced TLR4 expression and inflammatory responses in macrophages (81). Besides, macrophages have been shown to play a central role in the inflammatory phase of tissue repair, such as the removal of dead cells, debris and pathogens. The dynamic plasticity allows them to mediate tissue damage and repair functions. For instance, LPS-pretreated UCMSC-derived exosomes can uniquely express miR let-7b, and upregulate of the expression of anti-inflammatory cytokines and promote M2 macrophage activation. Let-7b regulated macrophage polarization by downregulating TLR4/NF-κB/STAT3/AKT signaling pathway (82). In another study, miR-223 derived from BMSC-exosomes regulated macrophage polarization by targeting pknox1, suggesting that macrophage M2 polarization may accelerate wound healing *via* miR-223 (83). MiR-19b, derived from ADSC exosomes, were significantly increased in recipient cells and were able to decrease inflammatory CCL1 levels acting through TGF-β pathway in H_2_O_2_ pretreated HaCaT cells (84).

#### Exosomal miRNAs on anti-scarring

Skin fibrosis results from poor wound healing following severe tissue damage such as severe burns, trauma, and major surgery. Pathological skin fibrosis tends to cause scarring. Dysregulation of each stage of wound healing, including the inflammatory, proliferative and remodeling stages, can lead to skin fibrosis (85). In vivo, ADSC derived exosomes over-expressing miR-29a inhibited the proliferation, migration, fibrosis and scar hyperplasia of human hypertrophic scar fibroblasts after scald wound in mice by targeting TGF-β2/Smad3 signaling pathway (86). Besides, ADSC exosomal miR-192-5p reduced pro-fibrotic protein levels and ameliorated hypertrophic scar fibrosis in mice through the IL-17RA /Smad axis (87).

Myofibroblasts aggregation also results in excessive scarring. A study examined UCMSC exosomal miRNAs by high-throughput sequencing and found that a group of specific miRNAs (miR-21, miR-23a, miR-125b and miR-145) played a key role during myofibroblast formation. In vivo and *in vitro* studies have validated that these miRNAs inhibited α-smooth muscle actin formation and collagen deposition through suppressing TGFβ/Smad2 axis (88).UCB exosomes were identified to contain abundant miRNAs. MiR-21-5p and miR-125b-5p from UCB exosomes were found essential for TGF-β1-induced anti-myofibroblast differentiation in human dermal fibroblasts by repressing receptor type II (TGFBR2) and TGF-β receptor type I (TGFBR1) respectively (17).

### Stem cell derived exosomal lncRNAs and wound healing

Stem cell-derived exosomal lncRNAs promotes wound healing in cell proliferation, migration, angiogenesis, anti-inflammatory and anti-fibrosis (see Table 2).

**Table 2:** Stem cell-exosome derived lncRNA/cirRNA-mediated regulation of wound healing.

Exosome derived lncRNAs/circRNA	Donor cells	Recipient cells	Potential target factor	Functions	References
lncRNA MALAT 1	ADSCs	Fibroblasts	–	Enhance cell migration	(89)
lncRNA MALAT 1	ADSCs	HaCaT and fibroblasts	miR-124 Wnt/β-catenin↑	Promote cell proliferation, migration	(90)
lncRNA H19	ADSCs	Fibroblasts	miR-19b↓ SOX9↑ Wnt/β-catenin↑	Accelerate fibroblasts proliferation, migration and invasion	(91)
lncRNA H19	BMSCs	Fibroblasts	miR-152-3p↓ PTEN↑ PI3K/AKT↓	Prevent the apoptosis and inflammation of fibroblasts	(92)
lncRNA KLF3-AS1	BMSCs	HUVECs	miR-383↓ VEGFA↑	Accelerate the proliferation, migration and tube formation of HUVECs Inhibit the apoptosis of HUVECs challenged by high glucose	(93)
lncRNA GAS 5	ADSCs	Fibroblasts	TLR-7↓	Modulate inflammation and accelerate the healing of chronic recalcitrant wounds	(94)
circRNA 0000250	ADSCs	EPCs	miR-128-3p↓ SIRT1↑	Enhance autophagy, reduce apoptosis of EPCs, facilitate skin angiogenesis	(96)

ADSC, adipose-derived mesenchymal stem cell; BMSC, bone marrow-derived mesenchymal stem cell; EPCs, endothelial progenitor cells; PTEN, phosphatasetensin homolog; ↑, upregulated; ↓, downregulated.

The lncRNA MALAT 1, a lncRNA abundant in exosomes from ADSCs, was validated to have the function in promoting human dermal fibroblasts migration and ischemic wound healing (89). Another study demonstrated that MALAT1-enriched exosomes derived from ADSCs promoted cell proliferation, migration and apoptosis in H_2_O_2_ induced HaCaT and human dermal fibroblasts by targeting miR-124 and activating the Wnt/β-catenin pathway (90).

LncRNA H19 (H19) was one of the first discovered lncRNAs and was found enriched in MSC-exosomes. ADSC exosomes inhibited the expression of miR-19b *via* lncRNA H19, thereby upregulating SOX9 to activate the Wnt/β-catenin pathway, resulting in accelerating the proliferation, migration and invasion of human skin fibroblasts. In vivo experiments also confirmed that exosomes from ADSCs promoted mouse skin wound healing through H19 (91). In a streptozotocin-induced diabetic foot mouse model, BMSCs-derived lncRNA H19 transferred to fibroblasts *via* exosomes, prevented fibroblasts apoptosis and inflammation by attenuating miR-152-3p-mediated PTEN repression to improve diabetic wound healing (92).

Another lncRNA KLF3-AS1 from BMSC exosomes induced angiogenesis to promote wound healing in diabetic skin. KLF3-AS1 accelerated the proliferation, migration and tube formation of HUVECs, while inhibited the apoptosis of HUVECs challenged by high glucose. In vivo, exosomes from BMSCs over-expressing KLF3-AS1 also promoted skin wound healing in diabetic mice through increasing angiogenesis, and decreasing inflammation and miR-383 expression (93). Besides, LncRNA GAS 5, which is highly enriched in exosomes from ADSCs, was found to attenuate LPS induced inflammation in human skin fibroblasts, indicating it may have a role in promoting healing of chronic recalcitrant wounds (94). HOTAIR, a long noncoding RNA from extracellular vesicles of BMSCs, was confirmed to participate in promoting angiogenesis and wound healing in diabetic db/db mouse (95).

### Stem cell derived exosomal circRNAs and wound healing

The roles of circRNAs in wound healing have also received increasing attention because studies have shown that circRNAs involved in the physiological process of wound healing (see Table 2). CircRNAs are crucial in regulating different disease microenvironments. ADSC-exosomes containing high concentrations of mmu_circ_0000250 facilitated the recovery of endothelial progenitor cells (EPCs) function by enhancing autophagy, and reducing apoptosis of EPCs under high glucose conditions. In diabetic mouse wounds, mmu_circ_0000250-enriched exosomes facilitated skin angiogenesis and inhibited apoptosis through autophagy activation *via* inhibiting miR-128-3p and upregulating SIRT1 (96). Circ-Gcap14 from hypoxic preconditioned ADSCs increased the expression of angiogenic growth factors and accelerated diabetic wound closure by inhibiting downstream miR-18a-5p and promoting the expression of HIF-1α in a mouse wound model (97).

### Stem cell derived exosomal mRNAs and wound healing

Current studies have confirmed that mRNAs are enriched in exosomes, can be transferred to recipient cells and perform coding functions. Few studies have focused on the contribution of exosomal mRNAs in wound healing. A study revealed that microvesicles derived from UCMSCs improved the dedifferentiation and proliferation of damaged tubular cells by transferring hepatocyte growth factor (HGF) mRNAs and enhancing HGF synthesis, thereby accelerating renal regeneration and delaying fibrosis (98). Deregibus et al. first reported that endothelial progenitor cell-derived microvesicles improved angiogenesis through transfered mRNAs associated with PI3K/AKT and endothelial NOS signaling pathways (99). Future research may focus on how to transport functional mRNAs to encode active molecules for wound healing.

## Conclusion and perspective

In recent years, there have been a large number of studies confirming that exosomes derived from different stem cell types are effective for wound healing (15, 79, 81, 100, 101). Since the targets of exosomes on recipient cells are not well-defined, the investigation of their molecular mechanisms is necessary. The skin wound healing process is a precisely regulated, therefore, the functions of the transcriptomes of exosomes on skin wound healing are complex. At present, the majority of the research work concentrated on how miRNAs from the stem cell derived exosomal transcriptomes act on fibroblast proliferation and migration in wound healing, and yet a few studies aimed at lncRNAs, circRNAs and mRNAs. This article reviews the major effects of mRNAs, miRNAs, lnc RNAs, circRNAs, and other stem cell derived exosomal transcriptomes on wound healing. Most of these exosomal RNAs were derived from mesenchymal stem cells, especially ADSCs and BMSCs. Although these RNAs are from different sources, their functions are nearly identical.

Exosomes are abundant in RNAs, including coding or non coding RNAs (ncRNAs). It is worth exploring whether RNAs interact with each other. The prevailing hypothesis is competitive endogenous RNA (ceRNA) hypothesis regarding the interplay between RNAs currently. The current research on stem cell derived exosomes and ceRNAs is still lacking. Han et al. investigated the interaction between differentially expressed lncRNAs, miRNAs and mRNAs during wound healing in normal individuals. After analyzing the ceRNA network, four up-regulated lncRNAs (MEG8, MEG3, MIR181A1HG, MIR4435-2HG) were found express during wound healing. MEG8/MEG3 may regulate fibroblast proliferation, differentiation and apoptosis *via* hsa-miR-296-3p/miR-6763-5p (102).With this lead, more studies regarding the interaction between ceRNA and other RNAs in wound healing are warranted.

To date, there have been a number of studies on the clinical application of ncRNAs which served as biomarkers for judging disease progression and prognosis of patients (103–105). However, the application of stem cell-derived exosomal transcriptomes remains at the basic research stage, and some urgent concerns need to be solved before clinical translation. For example, which source of exosomal transcriptomes is with the most potent efficacy? Which ncRNA plays the major regulatory role? Do lncRNAs alone or co-working with other exosomal components such as lipids and proteins participate in wound healing? How to improve the yields of specific RNAs from exosomes? Although the above challenges exist, with the rise of gene editing technology and the continuous development of RNA delivery techniques, it is foreseeable that stem cell derived exosomal transcriptomes can be widely used in the field of regenerative medicine in the near future.
